# Marked Difference in the Conformational Transition of DNA Caused by Propanol Isomer

**DOI:** 10.3390/polym12071607

**Published:** 2020-07-19

**Authors:** Yue Ma, Yuko Yoshikawa, Hidehiro Oana, Kenichi Yoshikawa

**Affiliations:** 1 Faculty of Life and Medical Sciences, Doshisha University, Kyotanabe 610-0394, Japan; gdmymayue@gmail.com (Y.M.); yoshi2989r@gmail.com (Y.Y.); 2 Department of Mechanical Engineering, The University of Tokyo, Tokyo 113-8656, Japan; oana@mech.t.u-tokyo.ac.jp

**Keywords:** genomic DNA, 1- and 2-propanol, high-order structure, single-molecule observation, intrachain phase segregation

## Abstract

We measured the changes in the higher-order structure of DNA molecules (λ phage DNA, 48 kbp) at different concentrations of 1- and 2-propanol through single-molecular observation. It is known that 2-propanol is usually adapted for the procedure to isolate genomic DNA from living cells/organs in contrast to 1-propanol. In the present study, it was found that with an increasing concentration of 1-propanol, DNA exhibits reentrant conformational transitions from an elongated coil to a folded globule, and then to an unfolded state. On the other hand, with 2-propanol, DNA exhibits monotonous shrinkage into a compact state. Stretching experiments under direct current (DC) electrical potential revealed that single DNA molecules intermediately shrunk by 1- and 2-propanol exhibit intrachain phase segregation, i.e., coexistence of elongated and compact parts. The characteristic effect of 1-propanol causing the reentrant transition is argued in terms of the generation of water-rich nanoclusters.

## 1. Introduction

In biochemical and biomedical studies, alcohol precipitation is widely used to purify or concentrate nucleic acids [1,2,3,4,5,6,7,8,9,10,11,12,13,14,15,16,17,18]. 2-Propanol (isopropyl alcohol) is often used to isolate DNA molecules from cells through precipitation [4,6,7,9,10]. On the other hand, 1-propanol is not adopted as the solvent for DNA precipitation. Currently, 1-propanol is regarded as a useful solvent in the pharmaceutical industry, mainly for resins and cellulose esters [19,20]. Although 1- and 2-propanol have been adapted for different purposes, the underlying physicochemical difference between these isomers seems to have not yet been clarified. Previous research tried to explain this via the solubility of DNA in different solutions [21,22]. However, there is still no clear physicochemical explanation for why 2-propanol is desirable [4,6].

To shed light on the large differences in effects on DNA between 1- and 2-propanol, here we performed a study on the higher-order structural change in DNA molecules, together with the measurement of the secondary structure. The high-order structural transition of DNA between elongated coil and compact globule states, the so-called coil–globule transition, is one of the central problems in the fields of biochemistry, biophysics, and soft matter physics. Many studies have examined this problem from both theoretical and experimental approaches [23,24,25,26,27,28,29,30,31,32,33,34,35,36,37,38]. Over the past couple of decades, it has been established that large DNA above the size of several tens of kilo base pairs (kbp) exhibits unique conformational characteristics, including the occurrence of discrete, or on/off, transition between the elongated coil and compact globule states on individual DNA molecular chain, as demonstrated by single-molecule observation in bulk solutions using fluorescence microscopy [28,32,35,39]. In the present study, it was found that with an increasing concentration, the conformational transition of DNA exhibits marked difference in 1- and 2-propanol solutions. In addition, it was confirmed that from the single DNA measurements by the application of DC electrical potential, intrachain phase segregation was showed on DNA molecules with the intermediate shrunken state.

The results of present study will provide reasonable explanation for why 2-propanol solution is more frequently used to retrieve genomic DNA molecules [4,7,9,21]. It is also expected that the generation of intrachain phase segregation on a single DNA observed in the present study may provide a useful insight into the intrinsic properties of genome-sized DNA molecules.

## 2. Materials and Methods

### 2.1. Materials and Preparation of Samples

The bacteriophage λ-DNA (48 kbp) was purchased from Nippon Gene (Toyama, Japan). DNA samples were dissolved in a propanol–water solution with a final concentration of 30 μM in nucleotide units. A fluorescent cyanine dye, YOYO-1 (quinolinium, 1, 1′-[1, 3-propanediyl-bis [(dimethylimino)-3, 1-propanediyl]] bis [4-[(3–methyl-2(3H)-benzoxazolylidene)-methyl]]-tetraiodide), was purchased from Molecular Probes, Inc. (Eugene, OR, USA). The antioxidant 2-mercaptoethanol (2-ME) and 1- and 2-propanol were purchased from Wako Pure Chemical Industries (Osaka, Japan).

### 2.2. Observation of the Higher-Order Structure of DNA by Fluorescence Microscopy

YOYO-1 (final concentration: 1 μM) was added to the DNA solution, together with 4(*v*/*v*)% 2-ME before observation. Fluorescence DNA images were captured using an Axiovert 135 TV microscope (Carl Zeiss, Oberkochen, Germany) equipped with an oil-immersed 100× objective lens and recorded on DVD using an EBCCD camera (Hamamatsu Photonics, Hamamatsu, Japan). The recorded videos were analyzed using VirtualDub (written by Avery Lee) and ImageJ software (National Institute of Mental Health, Bethesda, MD, USA). All observations were carried out at around 25 °C.

### 2.3. Circular Dichronism (CD) Measurements

The CD spectra of λ-DNA were measured with a CD spectrometer (J-820, JASCO, Tokyo, Japan). Measurements were performed at a scan rate of 100 nm/min, and 2000 μL of each sample was tested at around 25 °C. The cell path length was 1 cm, and CD spectra were obtained as the accumulation of three scans. The analysis of the observed CD spectral change was performed with reference to past studies [40,41,42,43,44,45].

## 3. Results

### 3.1. Higher-Order Structural Change of DNA Molecules with Alcohol

**Figure 1:**
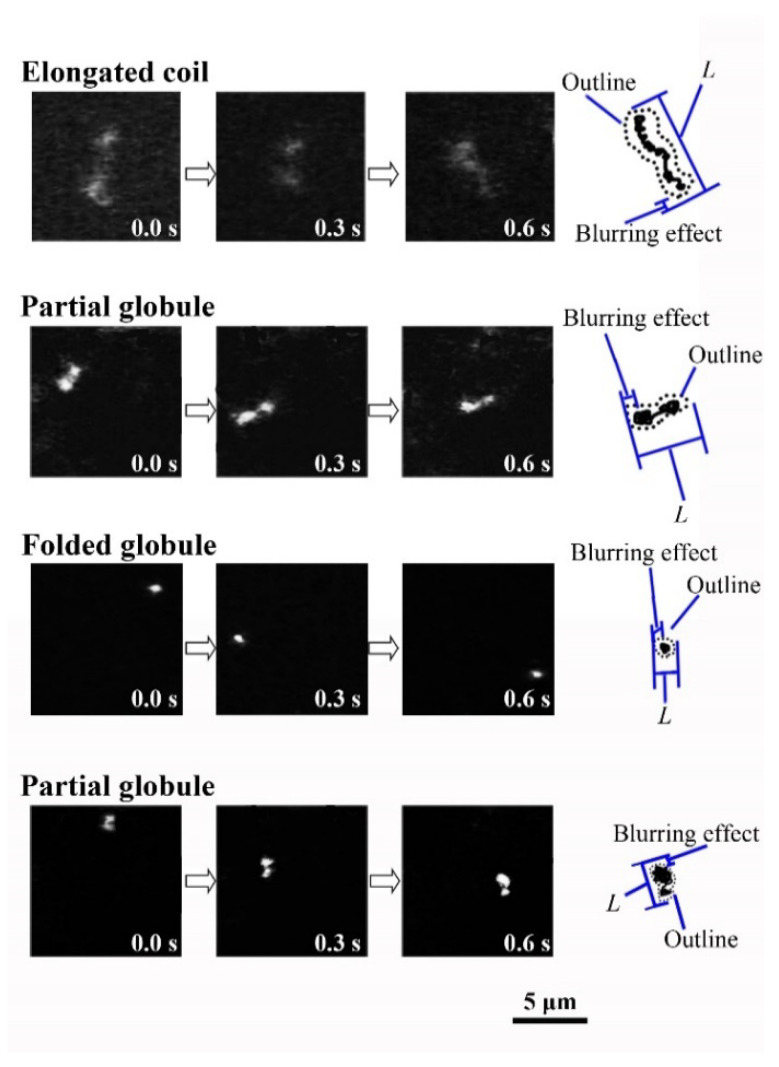
Fluorescence images of λ-DNA molecules (48 kbp) exhibiting Brownian motion in 1-propanol solutions; from top to bottom: 0, 50, 60, and 75(*v*/*v*)%.

Figure 1 exemplifies the fluorescence microscopic observations on single λ-DNA molecules at different 1-propanol concentrations, where DNA exhibits Brownian fluctuations. Here, it is noted that DNA exhibits folding transition from elongated coil to compact globule with the increase of 1-propanol concentration up to 60(*v*/*v*)%. On further increase in the 1-propanol concentration to 70(*v*/*v*)%, DNA exhibits unfolding transition to a swelled state, i.e., DNA undergoes reentrant transition. Similar behavior of the reentrant transition of DNA conformation was also observed in ethanol solution [28].

**Figure 2:**
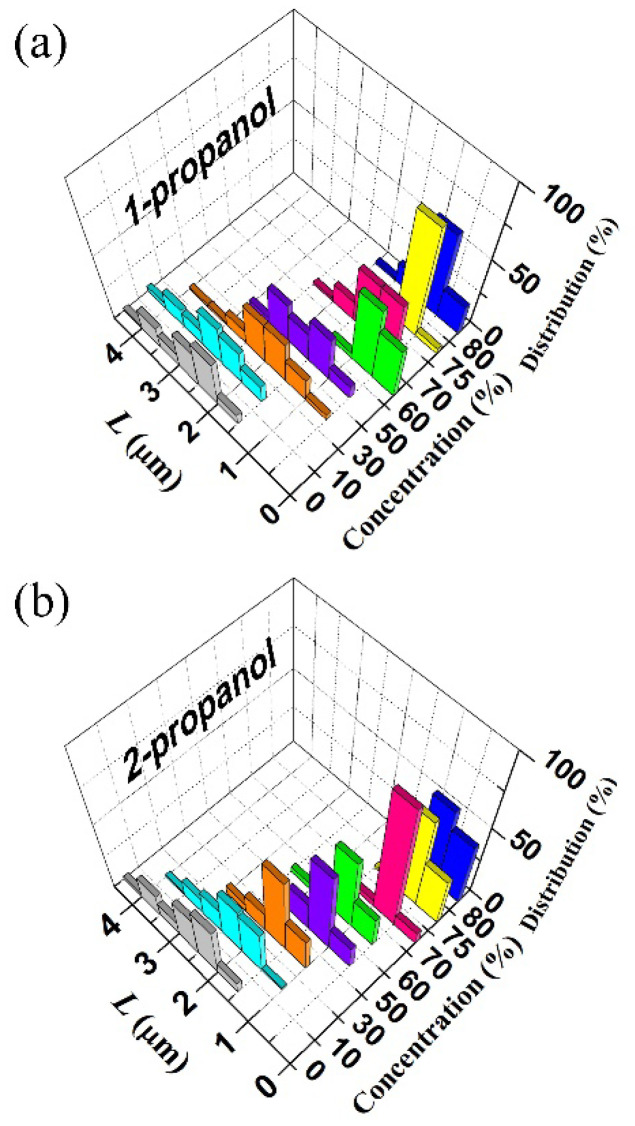
Histogram of the long-axis lengths, *L,* of λ-DNA molecules at different concentrations of (**a**) 1-propanol and (**b**) 2-propanol. Each color represents a distribution with different concentration of propanol.

To evaluate the conformational change of λ-DNA molecules in a semi-quantitative manner, we measured the long-axis length, *L* [28,32,34,35,46,47]. The histogram in Figure 2 shows the distribution of the long-axis length of DNA molecules. (The changes of the average long-axis lengths, <*L*>, of the corresponding data are shown as a graph in Figure 2a). For DNA molecules in different concentrations of 1-propanol, one minimum appeared at 60(*v*/*v*)%, suggesting the occurrence of reentrant folding-unfolding transition on DNA conformation. On the other hand, for the case of 2-propanol, the average long-axis length of DNA decreased monotonously, i.e., DNA stayed as a folded globule around 70–80(*v*/*v*)% of 2-propanol.

**Figure 3:**
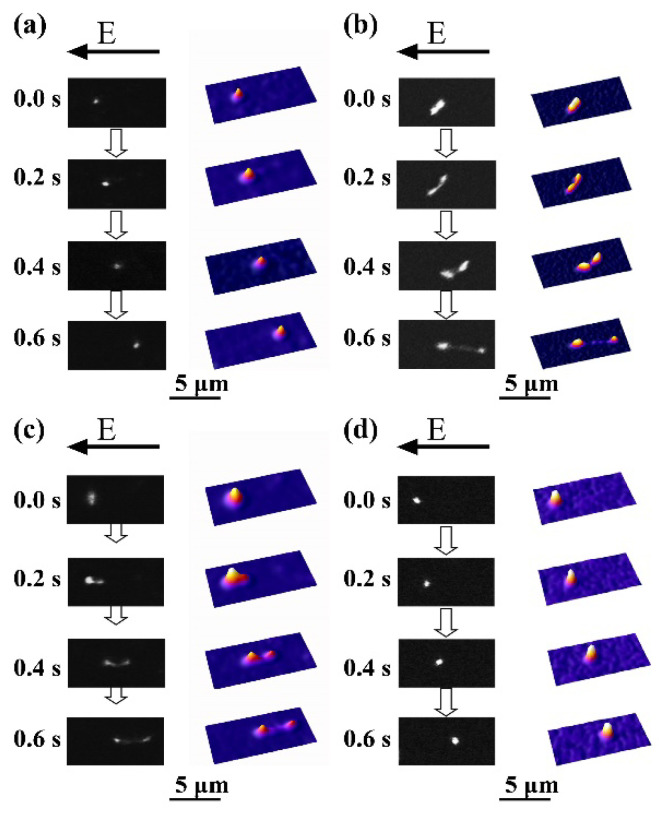
Change in the conformation of a single λ-DNA molecule after the application of a DC electric field, E, of ca. 10 V/cm. Left, fluorescence images; Right, quasi-three-dimensional representations on the fluorescence intensity distribution. (**a**) The folded globular molecule in 60(*v*/*v*)% 1-propanol solution; (**b**) the partial globular molecule in 70(*v*/*v*)% 1-propanol solution: there is clear segregation of the globular and coil parts; (**c**) The partial globular molecule in 60(*v*/*v*)% 2-propanol solution; (**d**) the folded globular molecule in 70(*v*/*v*)% 2-propanol solution.

In order to gain further insight into the DNA conformation, we carried out the fluorescence microscopic observations on single DNA molecules by the application of a DC electric field of ca. 10 V/cm [32]. It has been argued in a past research that the negative charge along the DNA chain is almost neutralized in the globule state [48]. Thus, the intrinsic electrophoretic mobility of coil DNA is much larger than that of globule DNA under a DC electric field, which causes the elongation of the coil part in partially globular DNA [32]. For compact folded states, as in the DNA samples with 60(*v*/*v*)% 1-propanol (Figure 3a) and with 70(*v*/*v*)% 2-propanol (Figure 3d), the bright spot stays stable even under the application of an electric field, confirming that the molecule is in a tightly packed state, i.e., as a folded globule. In contrast, for the intermediately swelled DNA molecules with 70(*v*/*v*)% 1-propanol (Figure 3b) and with 60(*v*/*v*)% 2-propanol (Figure 3c), the bright spots tend to be separated and accompanied by the elongation of the interconnected coil part, revealing that folded compact and elongated coil parts coexist along individual single DNA molecules, clearly revealing the appearance of the phase-segregated state for the intermediate shrunken DNA [32,35,39].

### 3.2. Analysis of the Brownian Motion of Single DNA

**Figure 4:**
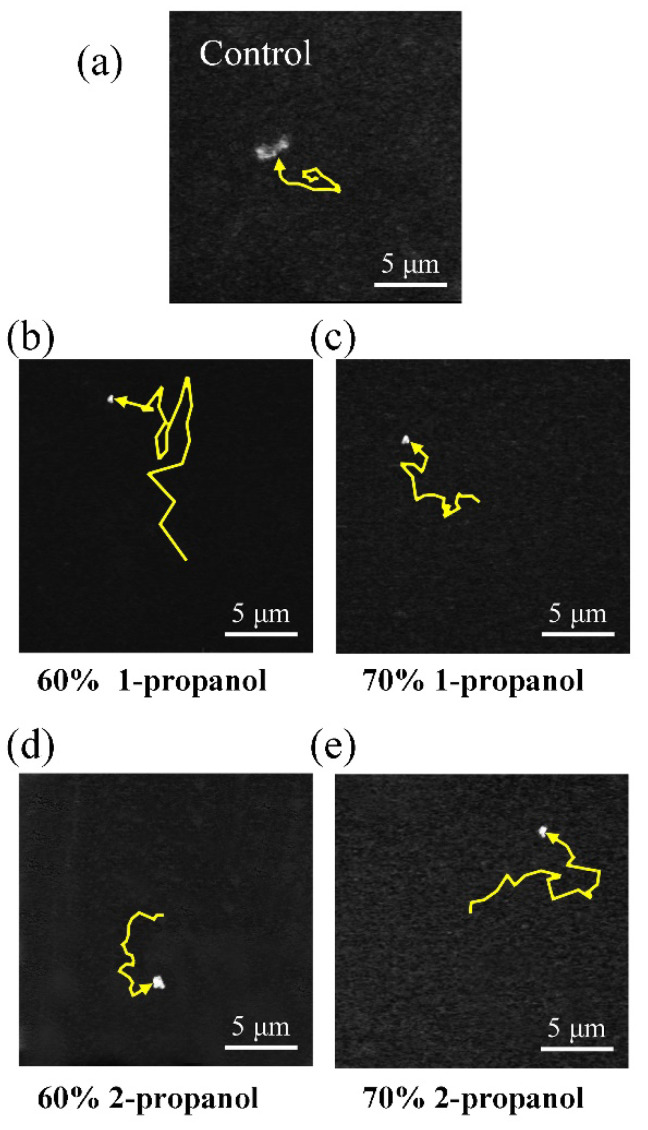
Trajectories of the center of mass of individual molecules in (**a**) solution without propanol, (**b**) 60(*v*/*v*)% 1-propanol solution, (**c**) 70(*v*/*v*)% 1-propanol solution, (**d**) 60(*v*/*v*)% 2-propanol solution, and (**e**) 70(*v*/*v*)% 2-propanol solution, as observed by fluorescence microscopy for 3 s.

In Figure 2 we have shown the change in the long-axis length, *L*, on individual DNA molecules exhibiting translational and intrachain Brownian motion. Owing to the time-dependent fluctuation in the DNA fluorescence image in addition to the blurring effect (approximately 0.3 μm), the information of the apparent long-axis consisted of a relatively large experimental error. Thus, the quantitative analysis of the translational Brownian motion was performed on individual DNA molecules [46,47] in order to evaluate their hydrodynamic radius [49,50]. Figure 4 exemplifies the trajectories of the center of mass of individual single DNA, indicating a rather significant difference in the translational Brownian motion that depended on the solution compositions. The folded compact DNA molecule in 60(*v*/*v*)% 1-propanol solution apparently exhibits a significantly larger fluctuation than the other samples. The molecule without alcohol is less active than the DNA molecules in other solutions with alcohols. From the motion trails of the center of mass of a single DNA molecule during the thermal fluctuation, we calculated the mean square displacement and evaluated the diffusion constant *D* for each DNA molecule using Equation (1) [46]:

(1) $$\langle {\left(r\left(t\right)-r\left(0\right)\right)}^{2}\rangle =4Dt+At^{2}$$

where $r\left(t\right)=\left(r_{x},\;r_{y}\right)$ is the position of the center of mass for a DNA, $\langle {\left(r\left(t\right)-r\left(0\right)\right)}^{2}\rangle$ is the mean square displacement, and *A* is a numerical constant related to convective flow. The hydrodynamic radius $R_{H}$ is calculated from *D* based on the Stokes–Einstein relation [47]:

(2) $$R_{H}=\frac{k_{B}T}{6\pi \eta_{s}}\;\cdot \;\frac{1}{D}$$

where $k_{B}$ is the Boltzmann constant and $\eta_{s}$ is the viscosity of the solvent at 298 K. The viscosity of the solvent was referred from the literature [51].

**Figure 5:**
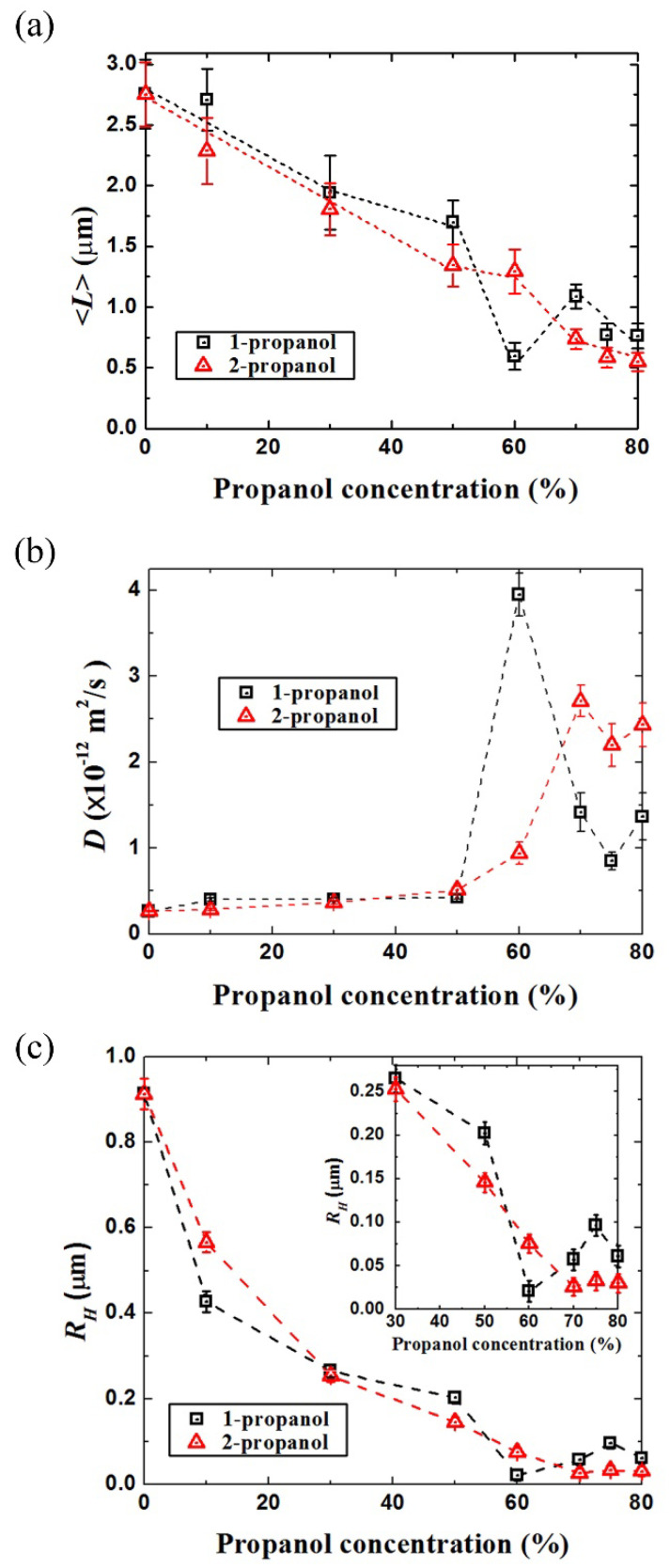
(**a**) The average long-axis length of DNA molecules in different alcohol solutions; (**b**) diffusion constant of DNA molecules (which indicate the difference in Brownian motion of molecules in different solutions), *D*, at different propanol concentrations; (**c**) hydrodynamic radius of DNA molecules, *R_H_*, at different propanol concentrations (*R_H_* in high concentrations of propanol was enlarged on the top of right side).

As shown in Figure 5c, the change in the hydrodynamic radius of DNA molecules, $R_{H}$, corresponds well to the change in their long-axis length. For DNA molecules in different concentrations of 1-propanol solutions, $R_{H}$ decreased from 0.91 μm to 0.02 μm as the concentration of 1-propanol increased to 60(*v*/*v*)%. A minimum $R_{H}$ (0.02 μm) also appeared at 60(*v*/*v*)%. On the contrary, by changing the concentrations of 1-propanol from 60(*v*/*v*)% to 75(*v*/*v*)%, $R_{H}$ increased from 0.02 μm to 0.10 μm. This demonstrated that DNA molecules swelled or unfolded above 70(*v*/*v*)%. By considering $R_{H}$ as the one-dimensional size of DNA, the relative ratio of (0.10 μm)/(0.02 μm) = 5.0 was found to correspond to the volume ratio with (5.0)^3^ ≈ 10^2^, indicating a swelling ratio of hundred times. In 2-propanol solutions, the $R_{H}$ of DNA decreased from 0.91 μm to 0.03 μm in a monotonous manner as the concentration of 2-propanol increased. Thus, the changes in the diffusion constant shown in Figure 5b clearly reveals a marked difference in the high-order structure of DNA with different propanol solutions in terms of the reliable physicochemical parameter of $R_{H}$.

### 3.3. Secondary Structure of DNA Molecules in Alcohol Solutions

**Figure 6:**
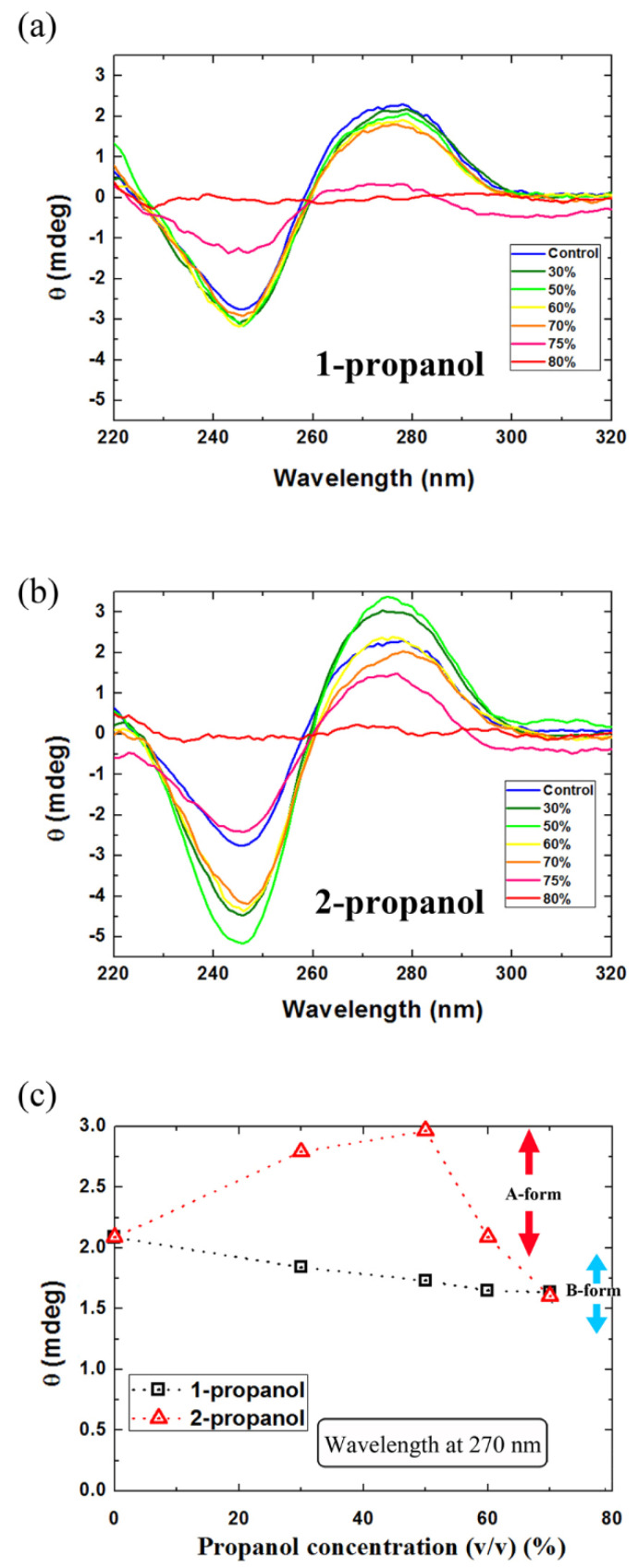
CD spectra of DNA (λ-DNA, 30 μM in nucleotide units) in (**a**) 1-propanol and (**b**) 2-propanol solutions; (**c**) degree of ellipticity (*θ*) of CD spectra of DNA samples at 270 nm.

As shown in Figure 6a, for DNA molecules in 1-propanol solutions, based on the positive band at around 275 nm and the negative band at around 245 nm, DNA maintained a B-like secondary structure from 0 to 70(*v*/*v*)% [28,42]. The spectra approached zero when the concentration of 1-propanol is higher than 70(*v*/*v*)%, which was attributed to the effect of precipitation accompanied by the condensation of DNA molecule. For samples in 2-propanol solutions (Figure 6b), the secondary structure changed to A-like form [28,42] from 30 to 60(*v*/*v*)%, since the positive band is higher and the negative band is lower than those for samples in 0(*v*/*v*)%. The secondary structure returned to B-like at around 70(*v*/*v*)% of 2-propanol. At a concentration higher than 75(*v*/*v*)%, the effect of DNA precipitation became non-negligible.

To clearly understand the observed changes in the CD spectra, the degrees of ellipticity (*θ*) at 270 nm are shown in Figure 6c. It is found that DNA molecules in 1-propanol only showed the B-like form before DNA deposition. On the other hand, in 2-propanol solution, both the A-like and B-like forms appeared when the concentration of 2-propanol was lower than the DNA deposition concentration.

## 4. Discussion and Conclusions

From the experimental data of the average long-axis length, <*L*>, the translational diffusion constant *D* and the hydrodynamic radius *R_H_*, as summarized in Figure 5, it becomes clear that the average long-axis length of DNA molecules in solution decreased as the concentration of 1-propanol increased to 60(*v*/*v*)%, then increased slightly and remained constant as the concentration continued to increase. For 2-propanol solutions, the average long-axis length decreased as the concentration increased, and then remained steady at a minimum value at high concentration.

Since 1-propanol molecules are straight-chained, like ethanol, they should exhibit similar polarity. The occurrence of the reentrant transition with ethanol and 1-propanol but not with 2-propanol may might be attributed to such a geometrical difference between the chemical structures of the alcohols. The secondary structure of DNA molecules retained a B-like form in 1-propanol solutions. However, in 2-propanol solutions, it changed to an A-like form and then back to a B-like form as the concentration of 2-propanol increased. Moreover, past research by our group on the secondary structure of DNA in ethanol solutions demonstrated that DNA showed B-, C- and A-like forms with an increase in the concentration of ethanol [28] The observed large difference in the effects of ethanol and propanol isomers on the DNA conformation is attributable to the difference in the nanostructure of a mixed solution between water and alcohol [52,53,54,55,56,57].

Past literatures have reported the formation of clusters in the aqueous solutions of alcohols [52,53,54,55,56,57,58,59]. The reentrant transition of the higher-order structure of DNA molecules is attributable to the association of water nanoclusters onto negatively charged phosphate groups along a double-stranded DNA molecule. Through this hydration effect by water nanoclusters, the phosphate groups of DNA tend to dissociate to negatively charged state by eliminating counter cations to the nearby environment. Thus, DNA molecule undergoes conformational transition from compact state onto a swelled state at higher concentrations of 1-propanol, similar to the reentrant transition of the higher-order structure of DNA depending on the concentration of ethanol [28]. As the next study, it may be of interest to examine the different manners of mixing states between the propanol isomers, where water-rich nanoclusters will be preferentially generated with 1-propanol as compared with the solution with 2-propanol. The present results suggested that the structural differences in 1- and 2-propanol, with linear or branched carbon skeletons, caused a significant difference in their ability to form water-rich nanoclusters. The parallel aligned molecules of 1-propanol are expected to deplete water molecules to generate the clusters.

In the present study, it was discovered that with an increase in the concentration of 1-propanol, DNA exhibits a reentrant transition, whereas 2-propanol caused a monotonous change in the DNA conformation. The difference in the physicochemical effects of these propanol isomers, as observed in the present study, may explain why the 2-propanol solution has been frequently adopted to retrieve genomic DNA molecules from living cells [4,7,9,21]. The monotonous conformational transition of 2-propanol indicates the efficient precipitation/elimination of DNA molecules from cellular extracts, whereas the reentrant transition with 1-propanol may cause undesirable effect for the purification. We have focused our interest on the effect of propanol isomers on DNA molecules and found the remarkable effect of a kind of micro/nano phase separation by using the term of nanoclustering. In the present study, we have adopted the solution conditions with low salt delivered from the available DNA specimens. Hereafter, it may be of interest to examine the effect of various salts on the behavior of DNA molecules in the presence of alcohols. It is highly expected that such extraordinary effects on DNA will inspire the study on the micro phase segregation concerning the stability and function of membraneless organelles [60].
